# Inositols’ Importance in the Improvement of the Endocrine–Metabolic Profile in PCOS

**DOI:** 10.3390/ijms20225787

**Published:** 2019-11-18

**Authors:** Anna Wojciechowska, Adam Osowski, Marcin Jóźwik, Ryszard Górecki, Andrzej Rynkiewicz, Joanna Wojtkiewicz

**Affiliations:** 1 Department of Pathophysiology, School of Medicine, Collegium Medicum, University of Warmia and Mazury, 10-082 Olsztyn, Poland; adam.osowski@uwm.edu.pl; 2 Department of Gynecology and Obstetrics, School of Medicine, Collegium Medicum, University of Warmia and Mazury, 10-082 Olsztyn, Poland; ginekologia@uwm.edu.pl; 3 Department of Plant Physiology, Genetics and Biotechnology, University of Warmia and Mazury, 10-082 Olsztyn, Poland; rigor@uwm.edu.pl; 4 Department of Cardiology and Cardiac Surgery, School of Medicine, Collegium Medicum, University of Warmia and Mazury, 10-082 Olsztyn, Poland; andrzej.rynkiewicz@uwm.edu.pl

**Keywords:** polycystic ovary syndrome, myo-inositol, d-chiro-inositol, androgen excess, ovulation problems, metabolic diseases

## Abstract

Polycystic ovary syndrome (PCOS) is one of the most common causes of infertility and metabolic problems among women of reproductive age. The mechanism of PCOS is associated with concurrent alterations at the hormonal level. The diagnosis assumes the occurrence of three interrelated symptoms of varying severity, namely ovulation disorders, androgen excess, or polycystic ovarian morphology (PCOM), which all require a proper therapeutic approach. The main symptom seems to be an increased androgen concentration, which in turn may contribute to different metabolic disorders. A number of papers have demonstrated the significant role of inositol therapy in PCOS. However, there is a lack of detailed discussion about the importance of myo-inositol (MI) and d-chiro-inositol (DCI) in reference to particular symptoms. Thus, the aim of this review is to present the effectiveness of MI and DCI treatment for PCOS symptoms. Moreover, the review is focused on analyzing the use of inositols, taking into account their physiological properties, together with the mechanism of individual PCOS symptom formation.

## 1. Introduction

In recent years, a large amount of attention from the scientific and medical community has been focused on the etiology and treatment of polycystic ovary syndrome (PCOS). It has been demonstrated that PCOS is the most common cause of infertility in women during reproductive age [1]. Among the current treatments for PCOS, first-line therapies can be distinguished, such as lifestyle modification or oral contraceptive pills, as well as treatments for patients with ovulation disorders, including ovarian hippocampal signal path block theory, the theory of leptin, or inositol treatment [2]. The mechanisms of action for most of these methods are directional—with more of an impact on some PCOS symptoms and less on others. There are many original and review papers that demonstrate the role of inositols in PCOS treatment [3,4,5], but without focusing on their effectiveness. This review presents a detailed discussion about the role of myo-inositol (MI) and d-chiro-inositol (DCI) in the management of women with PCOS in reference to particular symptoms. Moreover, the authors analyze the current data about the usage of inositols, taking into account their physiological properties. Thus, the aim of this paper is to demonstrate the importance of inositols together with the mechanism of individual PCOS symptom formation. The first part of the review introduces the topic, presenting the definition and pathogenesis of PCOS, as well as providing general information about inositols and their role in medical treatment. The next part describes the detailed pathophysiological mechanism of major PCOS symptoms and the significant role of MI and DCI in their treatment.

## 2. Polycystic Ovary Syndrome (PCOS)

### 2.1. Definition and Classification

Polycystic ovary syndrome is a heterogeneous endocrine disorder which affects 5–15% of the general population, depending on the ethnicity and the applied classification system [6]. Because of the symptoms’ heterogeneity and variability over time, it is difficult to clearly diagnose PCOS. The most common diagnostic criteria (Table 1) were defined by The European Society of Human Reproduction and Embryology and the American Society for Reproductive Medicine at Rotterdam, in a meeting in 2003 [7]. According to these arrangements, the diagnosis should be based on at least two out of three major clinical and metabolic abnormalities, including oligo-ovulation or anovulation, hyperandrogenemia or hyperandrogenism, and typical ovarian ultrasound features [8]. It is worth adding that polycystic ovarian morphology (PCOM) is not a necessary condition to state PCOS, and its presence alone does not establish a diagnosis [9]. Under the Rotterdam criteria, PCOS prevalence affects 18% of women of reproductive age [10]. Scientists also use the criteria listed by the Androgen Excess Society (AES) or the National Institutes of Health (NIH). According to these classification systems, women with PCOS should demonstrate different combinations of individual features related to PCOS. The main feature of the AES criteria is clinical or biochemical hyperandrogenism, which should be present together with oligo-/anovulation and/or polycystic ovaries [11]. However, chronic oligo-/anovulation, in the first place, combined with androgen excess, is characteristic in the NIH classification [12]. All of the diagnostic systems assume the exclusion of other disorders, which can be manifested by irregular menstrual cycles and/or androgen excess, such as Cushing’s syndrome, androgen-secreting tumors, increased prolactin (PRL), or luteinizing hormone (LH) [13].

### 2.2. Pathogenesis of PCOS

Despite many years of research, it is still difficult to determine the etiology of PCOS. Studies have suggested a strong genetic component, but the PCOS mechanism also has an environmental basis influenced by embryonic factors [14]. Genetic determination has been proven during studies on twin girls with a mother with PCOS, which showed that the twins had a double risk of developing the disorder until they reached their reproductive age [15]. The relatives of women with PCOS demonstrated also a higher frequency of glucose metabolism abnormalities and metabolic syndrome [16]. Moreover, the existence of specific genes responsible for PCOS features [14] and association telomere shortening with PCOS pathogenesis was demonstrated, especially in patients with a greater risk of metabolic comorbidities [17]. Environmental factors can be divided into prenatal factors, such as fetal developmental programming, and postnatal factors, which are associated with an unhealthy lifestyle and environmental pollutants [18]. It is known that during pregnancy, the fetus is particularly exposed to various signals from the mother. Furthermore, particular genes’ expression changes can be associated with an increased risk of metabolic and reproductive disorders in postnatal life [19]. Increased glucocorticoid and/or androgen exposure during critical periods of embryonic development can cause the developmental programming of PCOS in humans and animals [14,20]. Moreover, developmental programming by glucocorticoid excess may be related to PCOS and small-for-gestational-age newborns. In turn, these after-effects can result in a higher risk of cardiovascular diseases during adulthood [21]. On the other hand, the developmental programming by androgen excess can preferably lead to metabolic disorders, such as obesity, type 2 diabetes mellitus (DM), and insulin resistance in women [22]. Some papers have suggested that endocrine disrupting chemicals may affect PCOS development [23,24]. The exposure of pregnant rats to chemical mixtures caused PCOS-like symptoms as far as the third generation [25], while polychlorinated biphenyl exposure was associated with menstrual cycle abnormalities and the failure of implantation in in vitro fertilization (IVF) procedures [26]. It has also been shown that 25-hydroxyvitamin D in serum was lower in women with PCOS, which is associated with obesity and endocrine–metabolic disturbances [27]. However, vitamin D supplementation might improve menstrual frequency and metabolic disturbances, which proves the significant role of vitamin D in the pathogenesis of PCOS and PCOS-related complications [28].

**Figure 1:**
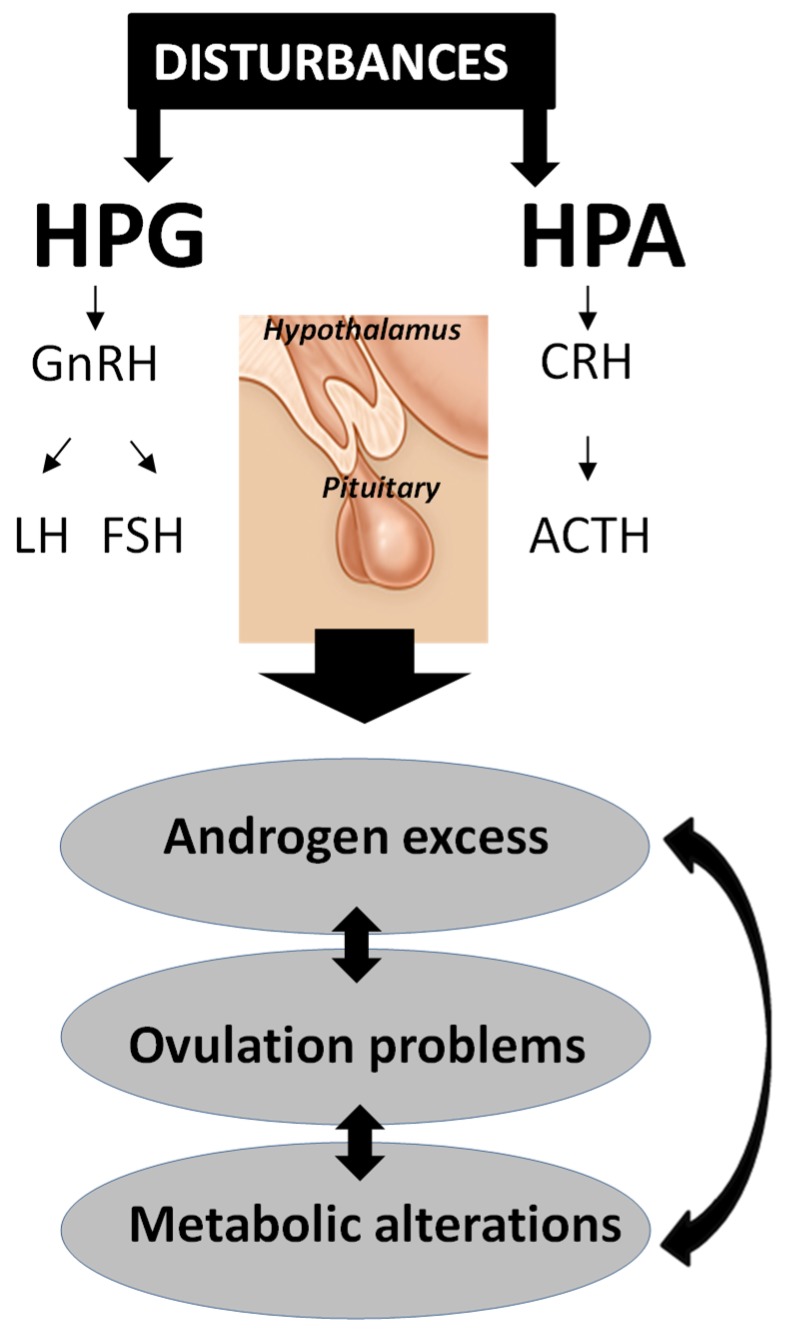
Disturbances at the hypothalamic–pituitary–gonadal (HPG) and hypothalamic–pituitary–adrenal (HPA) glands’ axes can lead to androgen excess, and consequently, anovulation and/or metabolic diseases, all three of which are the main symptoms of polycystic ovary syndrome (PCOS). GnRH—gonadotropin releasing hormone; CRH—corticotrophin releasing hormone; LH—luteinizing hormone; FSH—follicle stimulating hormone; ACTH—adrenocorticotropic hormone.

It should be considered that PCOS is the result of concurrent endocrinological alterations that interact with each other. The hypothalamic–pituitary–gonadal (HPG) and hypothalamic–pituitary–adrenal (HPA) glands’ axes function is essential in the PCOS mechanism; any disturbances at all of hormone levels can lead to androgen excess and/or anovulation (Figure 1). The endocrine profile of patients with PCOS is characterized by elevated plasma concentrations of ovarian and adrenal androgens, and thus higher levels of estrogens (mainly estrone). Physiologically, LH stimulates the conversion of cholesterol to androstenedione—the main precursor to testosterone (T) and estrogen synthesis—by ovarian theca cells. In the zona reticularis of the adrenal cortex, steroid production is stimulated by the adrenocorticotropic hormone (ACTH) and corticotrophin-releasing hormone (CRH), which are involved in the stress response. Androstenedione and T have an ovarian origin, while adrenal participation is demonstrated rather by the elevation of dehydroepiandrosterone sulphate. The increased pulsatility of hypothalamic gonadotropin-releasing hormone (GnRH) leads to an elevated frequency of LH pulses, as well as a LH/follicle-stimulating hormone (FSH) ratio. The higher stimulation of the theca cells by the LH level, reaching above 8–12 mIU/mL in PCOS, caused the increase of androgen biosynthesis, as well as follicular development disruption [29]. Furthermore, FSH affects the androgen aromatization and stimulates follicle maturation. Ovarian androgens stimulate follicle growth in the preantral and early antral stages, while their elevated concentration in the later antral stages can induce atresia. These hormonal changes have been observed together with the increase of PRL and insulin and have often appeared in overweight or obese patients, but also in the case of those with a normal weight or thin patients with PCOS [30,31]. It is well known that only about 1–2% of the bloodstream steroid hormones are unbound, and thus are biologically active. Apart from the extra glandular conversion, the elevation of the circulating steroids is caused by the reduced sex hormone-binding globulin (SHBG), which limits sex hormones’ bioavailability and function. On the contrary, increased androgen levels can regulate themselves by the reduction of hepatic SHBG synthesis, which results in a relative excess of free-circulating androgens. This mechanism, as well as the induction of peripheral conversion for dihydrotestosterone (DHT) by higher androgen levels, may cause hirsutism [31].

Studies have suggested that PCOS can begin very early in life, as evidenced by the specific precursors defined in childhood [32]. The daughters of women with PCOS before or during puberty had increased levels of T and anti-Müllerian hormone (AMH), as well as a higher number of follicles and ovarian volume compared with healthy women’s daughters [33,34]. Moreover, the PCOS daughters often had premature adrenarche, which increases the incidence of simultaneous PCOS [35,36]. Hormones associated with obesity in the cord blood of offspring with PCOS have been determined where a higher leptin level than insulin concentration was demonstrated [37]. However, hyperinsulinemia and decreased adiponectin levels were detected in these girls before puberty [33,34]. It is interesting that some changes have also been found in the sons of women with PCOS; for example, an elevated urinary T level in early puberty [38]. Considering that hormonal and metabolic changes precede reproductive abnormalities, the early diagnosis and monitoring of children from burdened families seems to be extremely important.

## 3. Inositol Characteristics

### 3.1. Isomers and Biosynthesis

Inositols are a group of natural polyols (sugars) that contain hydroxyl groups attached to a cyclohexane ring, and they belong to the class of organic compounds known as cyclohexanols. They are naturally present in food, including fruits, beans, grains, and nuts. Inositols, as components of cell membrane phospholipids, plasma lipoproteins, or the phosphate forms in the nucleus, are involved in many cellular processes, such as signal transduction, osmoregulation, or ion channel regulation in adults [39]. They are indispensable for proper fetal development as well as in the early postpartum period [40].

There are nine inositol stereoisomers, depending on the location of the hydroxyl groups; five of them (myo-, scyllo-, muco-, neo-, and d-chiro-inositol) occur naturally, while others (l-chiro-, allo-, epi-, and cis-inositol) are derived from myo-inositol (MI), which is actively synthetized by living cells. MI is unique among other forms of inositol, thanks to the single axial hydroxyl group located at the second carbon. Moreover, it is mostly found in nature; for example, in all eukaryotic cells [41]. The first step of MI biosynthesis is glucose phosphorylation to d-glucose-6-phosphate (G6P) by hexokinase. Then, G6P using myo-inositol-1L-phosphate synthase (MIPS) is converted to 1-l-myo-inositol-1-phosphate, which is dephosphorylated to free MI by the inositol monophosphatase [42]. MI synthesis occurs mostly in the human kidney, while MIPS is the rate-limiting enzyme of this process. l-chiro-inositol and d-chiro-inositol (DCI) are products of epimerizing hydroxyl groups of MI at the first and third carbon, respectively. The stereoisomer levels are regulated by an insulin-dependent epimerase, while the activity of this enzyme decreases drastically in the case of insulin resistance [41].

### 3.2. Mechanism of Action

It has been shown that the basis of the hyperinsulinemia is a defect in the inositol phosphoglycan (IPG) second messenger pathway [43]. The IPG production takes place at the cellular membrane level by glycosylphosphatidylinositol lipid hydrolysis. These molecules participate in the activation of the intracellular pathway responsible for oxidative and non-oxidative glucose metabolism and glucose uptake by glucose transporter type 4 from the extracellular environment [44,45]. The physiological ratio of MI and DCI differs between tissues, and both stereoisomers are regulated by an insulin-dependent epimerase whose activity is decreased in conditions of insulin resistance. Therefore, inositols participate in different insulin-dependent processes as insulin second messengers. MI is converted to an inositolphosphoglycan (IPG) insulin second messenger (MI-IPG) and is involved in cellular glucose uptake, whereas DCI is converted to an IPG insulin second messenger (DCI-IPG) and takes part in glycogen synthesis [46]. In the ovary, MI-IPG is involved in FSH signaling, whereas DCI-IPG is involved in insulin-mediated androgen production. Thus, disorders in the ovarian MI-DCI ratio might impair FSH signaling and worsen oocyte quality [47].

### 3.3. Clinical Relevance

Because of the wide occurrence and therapeutic properties of inositol isomers, they have successfully been used in therapies for many conditions. Apart from their effect in PCOS treatment, a number of studies have confirmed their participation in therapies of mental disorders, such as bipolar disorder, depression, panic attacks, obsessive–compulsive disorders, or eating disorders [48,49,50]. Moreover, MI can act as an important growth factor for human cells. The hormonal imbalance which is observed in women with PCOS results in an increased prevalence of osteoporosis [51]. However, MI and DCI can enhance osteogenesis and bone mineral density processes while inhibiting osteoclastogenesis [52]. Moreover, they are used in type 2 diabetes and insulin resistance treatment. MI induces translocation GLUT4 into the cell membrane and increases the cellular uptake of glucose in this way, whereas DCI takes part in insulin signaling by stimulating enzymes involved in the regulation of glucose metabolism [53]. Furthermore, DCI can improve the lipid and carbohydrate profile in pregnant women in the therapy of gestational diabetes. Both stereoisomers, due to their antioxidant, anti-inflammatory, and anti-cancer properties, are good therapeutic options for metabolic disorders, such as diabetes, hypertension, atherosclerosis, or allergic disease [53].

## 4. The Role of Inositol in PCOS Treatment

A number of studies have focused on the ability of inositols, especially MI and DCI, to enhance the ovulation rate and improve fertility in women with PCOS. Regardless of the adopted criteria of the PCOS diagnosis, the syndrome is associated with metabolic, hormonal, and reproductive aspects, which also require proper therapeutic treatment. The significant role of non-toxic MI and DCI in PCOS treatment is evident; however, their detailed application in relation to PCOS symptoms will be discussed in the following paragraphs.

Clinical data have demonstrated that MI and DCI, because of their insulin-sensitizing effect, successfully improved the metabolic and reproductive aspects of PCOS [5,54,55]. The physiological MI:DCI ratio and the relative amount of each stereoisomer are tissue-specific parameters which reflect the different physiological roles of inositols. It has been shown that MI increases the glucose cellular uptake and is involved in FSH activity, while DCI is crucial for glycogen synthesis and participates in the insulin-induced over-production of androgens in the ovary [56,57]. As a result of this, a high DCI level is observed in the glycogen storage tissues, such as the fat, liver, and muscle, while a low concentration occurs in energy-intensive tissues, such as the brain and heart [58]. It should be stressed that the DCI level is always lower than the MI in these systems. At a low dosage, DCI has restored normal insulin sensitivity in the typical insulin target tissues and reduced the circulating insulin and androgens [59]. These changes improve the ovarian cells’ function and ovulation frequency. The effect of MI on insulin metabolism occurs mainly at the ovary level, where it is highly concentrated. In this way, MI can directly affect ovarian functions, including steroidogenesis [60].

### 4.1. The Effect of Inositol in Androgen Excess

**Figure 2:**
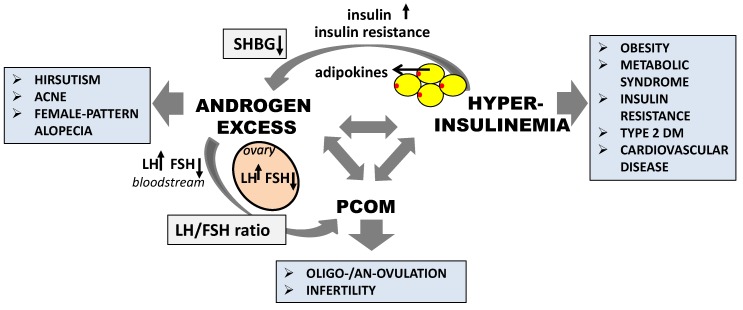
Selected interactions between the main symptoms of PCOS and their health consequences. LH—luteinizing hormone; FSH—follicle stimulating hormone; PCOM—polycystic ovarian morphology; DM—diabetes mellitus; SHBG—sex hormone-binding globulin.

It can be assumed that PCOS is a collection of symptoms arising from an excess of androgens [61]. Hyperandrogenism or excessive androgen levels are clinically manifested by features such as hirsutism, acne, and female-pattern alopecia. There is an inseparable connection between high androgen levels, anovulation, and insulin metabolism in women with PCOS (Figure 2). Hyperandrogenism is also associated with androgen synthesis and secretion by theca or *zona reticularis* cells [61]. However, hyperandrogenemia in patients with PCOS means elevated T and insulin concentrations in peripheral circulation, together with insulin resistance in the ovarian tissue. The circulating free androgen levels, especially T, are indirectly increased by two independent mechanisms. The excessive insulin concentration and insulin resistance stimulate the ovarian and adrenal secretion of the androgens and inhibit the hepatic production of SHBG [53]. The consequence of these hormonal changes can be premature follicular atresia, chronic anovulation, and gonadotropin imbalance, which was manifested by an increase in LH and decrease in FSH levels, as well as an LH:FSH ratio above 2.5 [62]. Moreover, ovarian androgen synthesis in PCOS, increased by insulin, seems to stimulate the granulose cells’ sensitivity to LH, as well as increasing the expression of the gene-encoding androgen-forming enzyme cytochrome P450c17α. Other studies have demonstrated that the theca cells of PCOS ovaries were more capable of transforming androgenic precursors to T; furthermore, the increased T production by the theca cells is more likely influenced by the excessive production of androgen precursors than by altered 17β-hydroxysteroid dehydrogenase activity [29].

A reduction of the free T (fT) level was observed after the administration of MI [63,64], DCI [65], or both [59], which can imply changes in the menstrual cycle and in fertility. Studies conducted by Regidor et al. demonstrated changes in the T level from 96.6 ng/mL to 43.3 ng/mL, and in the progesterone (P4) level from 2.1 ng/mL to 12.3 ng/mL, after MI and folic acid treatment in patients with PCOS [66]. Interesingly, Carlomagno et al. [67] demonstrated research comparing MI in two forms, powder and soft gelatin capsules, regarding their better bioavailability and absorption in the human body. The pharmacokinetic profile of gelatin MI was similar to that performed in the case of the MI powder at a three-times higher dose. Similarly, gel capsules containing both MI and DCI have been patented. It is worth adding that inositols in a gelatin capsule form can reduce the administered dose and the related side effects, as well as increasing their application in clinical practice, including PCOS [67]. Androgen excess as a PCOS symptom has been explained by two different mechanisms. First, a high androgen biosynthesis in PCOS could be the result of both insulin resistance and MI/DCI ratio disturbances in the ovary [47]. On the contrary, the increased androgen concentrations in women with PCOS could be related to a decreased MI:DCI relation, which, in turn, causes insulin resistance [68]. Therefore, there has been a reasonable amount of research in which the combination of both stereoisomers to treat PCOS symptoms has been used. Studies conducted by Januszewski et al. [69] have shown a significant fT, FSH, and LH reduction and SHBG plasma concentration increase in comparison with the values before MI and DCI administration, in a ratio of 10:1, respectively. These hormonal changes were associated with skin condition improvement during three months of treatment [69]. However, the combined administration of both compounds in a physiological plasma ratio of 40:1 seems to be the best choice of inositol therapy. Endocrine profile improvement in obese women with PCOS who were given MI and DCI, in a 40:1 ratio, compared to those given a placebo has been observed. Moreover, the fT and LH concentrations were decreased while estradiol (E2) and SHBG were increased in response to the combination of these compounds [59].

### 4.2. The Effect of Inositol in Ovulation Disorders and Infertility

PCOS as an endocrine disorder is a major cause of infertility and is associated with a lack of ovulation in women of reproductive age. In fact, 74% of patients with PCOS have cycles without ovulation. Moreover, approximately 30% of all infertility patients are non-ovulatory, and 90% of these conditions are caused by PCOS [2,70].

The significant role of MI administration for increasing the success rate of assisted reproduction techniques in PCOS is well known [71,72]. Data have been obtained in studies using MI and folic acid administered to infertile women over a time interval of two to three months [66]. This therapy resulted in an elevated rate of fertilization/pregnancy—around 15% of all of the studied women—and in better embryo quality. No significant side effects were observed among the patients, even at a dosage of 4000 mg MI per day [66]. Therefore, inositol treatment can help to restore spontaneous ovarian activity, such as spontaneous ovulation and menstrual cyclicity, and consequently, fertility, in many women with PCOS. It has also been suggested that MI excess in the ovary causes an increase in FSH sensitivity and improves fertilization rates and embryo quality in women with PCOS [73]. The results presented a smaller number of retrieved oocytes in the MI group, as well as a reduced risk of a hyperstimulation syndrome, which confirmed the advantages of using the MI to improve IVF protocols for patients with PCOS [66].

Gerli et al. have conducted a randomized, double-blind, placebo-controlled trial using a group of 283 women with PCOS. It was found that the circulating E2 concentration was elevated during the first week of inositol treatment, which was reflected in rapid follicular maturation [74]. A higher ovulation frequency and shortening of time to the first ovulation occurrence in the treated group was observed in comparison to the placebo group. The DCI or MI administration resulted in an almost two-fold increase in ovulation cases among women with increasing HDL cholesterol concentration and insulin sensitivity [75]. Treatment with MI at a dose of 2 g per day resulted in a reduced LH:FSH ratio and metabolic parameter decrease in obese women with PCOS [60,76]. However, other studies have shown that both MI and DCI, in a physiological plasma ratio of 40:1, can restore the hormonal features sooner than one-fold MI. The function of DCI in peripheral hyperinsulinemia reduction, and the role of MI in the improvement of the ovulatory function, explain the specific action of these compounds in a combined treatment. The effects of combined MI and DCI therapy versus MI monotherapy, with reference to ovarian function improvement, were compared by Nordio and Proietti [77]. Both MI and DCI positively affected the ovarian function in women with PCOS. Beneficial effects on the ovulation and metabolic parameters in PCOS by enhancing insulin sensitivity after DCI administration have been observed [78]. However, some papers have shown that DCI alone negatively affects the oocyte quality. The monotherapy using an increasing DCI dosage progressively deteriorated both the oocyte quality and ovarian response among women aged 40 who were diagnosed with PCOS, according to the Rotterdam criteria. There were no patients with insulin resistance and/or hyperglycaemia that participated in the studies. Therefore, the poor oocyte quality might be caused by the reduced energy metabolism [79]. This effect can be abolished by using MI in the therapy, because of its role in glucose cell uptake. This compound can also improve the ovary energy status and oocyte quality [80]. In connection with these reports, a treatment using both compounds, in the plasmatic ratio of 40:1, was affirmed by the Florence International Consensus Conference, on the use of MI and DCI in obstetrics and gynecology [58]. Moreover, Colazingari et al. [81] clearly showed an advantage of the combined treatment in a ratio of MI and DCI of 40:1 on the oocyte quality, comparing it to therapy using only DCI. Thus, combined therapy is the better choice for women with PCOS who are undergoing IVF with embryo transfer, in order to improve oocyte and embryo quality, as well as pregnancy rates.

### 4.3. The Effect of Inositol in Polycystic Ovarian Morphology

Another feature in diagnostic procedures which has been used since 2003 is PCOM ultrasonography; however, in this case, the exhibition does not automatically mean a PCOS diagnosis. Approximately 20–30% of women exhibit PCOM, but without other symptoms, they do not qualify as patients with PCOS [82]. During the evaluation of ovaries, it was suggested that attention should be paid to their sonographic appearance; in particular, the amount of antral follicles in each ovary [83]. In PCOS, 12 or more follicles in each ovary, with a 2–9 mm diameter, are observed. Moreover, the ovarian volume, which usually is increased in PCOS, as well as the increased AMH concentration are diagnostically relevant. AMH is produced by the granulosa cells of the preantral and small antral follicles in the ovary. In women with PCOS without ovulation, the granulosa cells synthesize AMH in concentrations much higher than in healthy ovulatory women as well as in ovulatory women with polycystic-appearing ovaries [84]. The level of AMH in the serum reflects the secretion only from the vascularised follicles. This hormone limits FSH-dependent processes, such as androgen to estrogen conversion, by granulosa cells and folliculogenesis. Thus, the increased AMH level inhibits follicle sensitivity to FSH, disrupts aromatase complex activity, and, in turn, adversely affects the final follicular maturation [79,85].

The ovarian phenotypes in women with PCOS were characterized by an increased conversion of MI to DCI, and a MI reduction in the follicles [56]. There was also a positive correlation between the volume of follicular fluid and its MI content, as well as the presence of matured and fertilized follicular oocytes [73]. Consequently, MI monotherapy has effectively improved the oocyte and embryo quality, and significantly affected the number of normal mature follicles or the amount of follicular fluid in a fertilized oocyte in patients with PCOS [69,77]. It has been demonstrated that DCI administration alone, at high doses, decreased the oocytes’ quality and ovarian response [79]. The best clinical results were achieved with the simultaneous use of MI and DCI during treatment [86]. Data, obtained after mice exposure of continuous light, have shown developed ovaries with morphological features typical of human PCOS and impaired gonad activity. Mice had a high ratio of theca/granulosa cell layer thickness (TGR), which authors identified as one of PCOS index. An increase of TGR in these mice correlated linearly with reproductive capability reduction. As a result, treatment of the mice with MI and DCI in a 40:1 ratio caused in ovaries complete recovery from PCOS symptoms to normal histological features as well as proper TGR ratio and fertility restoration [87].

Remarkable proposals have been made by Jiao et al. [88], who carried out research on ovarian tissue from patients with PCOS with irregular menstruation. They showed the genetic and epigenetic similarities between patients with ovarian cancer and irregular cycles. DNA hypomethylation, as well as mRNA and microRNA profiles in the ovaries of patients with irregular menstruation, converged with those from ovarian cancer tissue, which can increase the risk of this type of cancer in women with PCOS. Furthermore, multiple point mutations in the *BRCA1* and *MLH1* genes were demonstrated, which are directly related to ovarian cancerogenesis.

### 4.4. The Effect of Inositol in Metabolic Abnormalities

Insulin resistance and compensatory hyperinsulinemia are commonly observed dysfunctions in patients with PCOS [58]. It has been estimated that insulin resistance is characteristic of approximately 95% of obese patients, while this value is 60–80% for patients with PCOS [89]. Both disorders stimulated androgen secretion and suppressed SHBG synthesis, which led to hyperandrogenism, premature follicular atresia, anovulation, and gonadotropin imbalance [63]. Insulin resistance has been associated with the occurrence of many cardiometabolic disorders, such as obesity, impaired glucose tolerance, type 2 DM, dyslipidemia, hypertension, metabolic syndrome, and cardiovascular disease [32]. Lifestyle modifications, including diet control and physical exercises, seem to be extremely important and should be the first-line of treatment in overweight patients with PCOS [90]. It is even more important that excessive ovarian androgen production, as well as other symptoms, may be caused by endocrine changes occurring in the management of adipokines, which occur during weight gain and the development of inflammation in the adipose tissue [91]. It has been demonstrated that lifestyle changes in combination with drug therapy give positive results, not only in terms of body mass index reduction but also on the improvement of insulin resistance, menstrual disorders, and other PCOS symptoms [92]. Nybacka et al. [93] concluded that dietary and exercise administration decreased T and increased SHBG levels after four months of therapy. Moreover, 69% of women revealed menstrual cycle restoration. Systematic reviews and meta-analysis studies showed that lifestyle modification programs decrease glucose and insulin levels, suggesting a beneficial influence on overweight or obese women during PCOS treatment [94].

Most therapeutic strategies—for example, oestro-progestin pills or anti-androgenic compounds such as flutamide and finasteride—did not control the metabolic impairment of patients with PCOS. Inositols, through their insulin-sensitizing effect, were found to positively affect the metabolic parameters in women with PCOS. In PCOS, the limitations in the tissue availability or altered metabolism of inositol or IPGs, especially the IPG insulin second messenger pathway, may result in insulin resistance [95]. MI-IPG and DCI-IPG are involved in activating enzymes that control glucose metabolism. Therefore, inositol therapy is a good option in relation to the metabolic parameters, because of the cellular response improvement to the metabolic pathways.

Dyslipidemia is a common problem among the PCOS-related metabolic abnormalities. There have been observed lower concentrations of high-density lipoprotein cholesterol (HDL) and higher levels of low-density lipoprotein cholesterol (LDL) and triglycerides (TG) in women with PCOS, both being linked to insulin resistance and androgen excess [96]. Palomba et al. confirmed the above data in studies in a group of 150 healthy and non-obese women with PCOS [97]. Serum LDL and TG concentrations were significantly higher in women with PCOS than in healthy controls. Moreover, serum LDL and TG levels increased in both groups during pregnancy, although the changed values were more pronounced in patients with PCOS than in healthy controls, compared to those before pregnancy [97]. In the studies of Gerli et al. MI increased the HDL concentration. However, these changes were not observed in the morbidly obese group of patients with a BMI (body mass index) of above 37, which indicates an inverse relationship between BMI and efficacy of therapy [98]. Other studies have shown the metabolic profile of women with PCOS improved with the combined MI and DCI therapy. The levels of LDL, HDL, and TG were changed after MI and DCI treatment in a physiological ratio of 40:1 [99]. The administration of DCI decreased also the insulin response to glucose that was orally applied to obese women with PCOS [62]. Improvements in insulin resistance, blood pressure, and the concentration of plasma triglyceride were observed [65]. Significant effects on the metabolic and hormonal parameters were pronounced after treatment with MI and DCI in a 1:10 ratio. The study assumed an oral glucose tolerance test (OGTT) with the measurement of plasma glucose concentration after 1 and 2 h from a 75 g glucose load. In the patients with PCOS, diagnosed according to the Rotterdam criteria, body weight and insulin level reductions were demonstrated after six months of treatment [69]. The MI and DCI combination in a 40:1 ratio significantly reduced the fasting insulin, which helps to assess insulin sensitivity in young obese PCOS-affected women [59]. The synergistic activity of MI and DCI could cause an extension of insulin sensitivity in the liver and muscles and therefore a decrease in the circulating insulin.

The discovered insulin-sensitizing effect of MI proved to be important in the prevention of gestational diabetes mellitus (GDM) in patients with PCOS [100]. The risk of GDM reduction after MI supplementation during pregnancy has been demonstrated. Unfortunately, there was no effect on the fetal macrosomia or neonatal hypoglycemia associated with GDM. This may be due to the fact that, in human pregnancies, inositol is produced by the fetus and the placenta delivers sorbitol [101].

## 5. Conclusions

PCOS is definitely a multifactorial disease related to heredity, influenced by many environmental factors during gestation and adulthood. This literature review examined this in terms of inositols’ effectiveness in the treatment of individual PCOS symptoms (Table 2). The most effective results for all of the considered symptoms were obtained after the administration of both MI and DCI in a 40:1 ratio. This combined therapy should be used as the first-line of therapy in overweight patients with PCOS in order to effectively decrease the metabolic parameters and cause the clinical alteration of PCOS. Researchers have pointed out that the success of this combined inositol therapy depends on the functions of both compounds—a reduction in the peripheral hyperinsulinemia by DCI, and ovulatory function improvement by MI. Therefore, such an approach reduces the risk of metabolic syndrome and improves the endocrine profile and insulin resistance. Among the large number of alternative treatment methods for women with PCOS, the use of inositol seems to be an appropriate option, with a high efficiency and relatively few side effects.

Both stereoisomers have high drug tolerance and low toxicity, which, together with their common occurrence in plants, make these compounds widely used in medicine, even in pregnant women and children. However, the role of inositols in PCOS management still requires studies in larger research groups. Additionally, it would be worth expanding the research to the comparison of described inositols with other insulin-sensitizing compounds and testing new therapeutic combinations in relation to various PCOS symptoms.

## Figures and Tables

**Table 1:** Diagnostic criteria of polycystic ovary syndrome (PCOS) defined by The European Society of Human Reproduction and Embryology and the American Society for Reproductive Medicine at Rotterdam (Rotterdam), the Androgen Excess Society (AES) or the National Institutes of Health (NIH). The diagnosis is made after excluding other diseases with similar symptoms.

Diagnostic Criteria		
Rotterdam (at least two of three features)	AES (both features)	NIH (both features)
Oligo-/anovulation	Hyperandrogenism/hyperandrogenemia	Oligo-/anovulation
Hyperandrogenemia/hyperandrogenism	Oligo-/anovulation and/or Polycystic ovaries	Hyperandrogenism/hyperandrogenemia
Polycystic ovaries		

**Table 2:** The clinical studies that demonstrate the effects of myo-inositol (MI), d-chiro-inositol (DCI), or both in individual PCOS symptoms.

PCOS Symptom	Inositol	Effect	Authors
**Androgen excess**	**MI**	-total and free T level reduction	[64]
		-free T decrease; E2, SHBG increase	[59]
		-T level reduction	[63]
	**DCI**	-T level reduction and P4 level increase	[66]
		-free T decrease	[65]
	**both**	-free T reduction; SHBG increase	[69]
**Ovulation and fertility disorders; ovaries dysfunction**	**MI**	-better development of mouse embryos	[71]
		-restored spontaneous ovulation; elevated rate of fertilization/pregnancy	[66]
		-better embryo quality, no side-effects	[73]
		-increased FSH sensitivity, better fertilization rates and embryo quality; reduced LH:FSH ratio	[63]
		-restored spontaneous ovarian activity and fertility	[76]
		-higher ovulation frequency, shorter time to first ovulation	[98]
	**DCI**	-elevated E2; rapid follicular maturation	[74]
		-ovulation improvement	[78]
	**both**	-poor oocyte quality; worse ovarian response	[79]
		-increased ovulation cases	[75]
		-ovarian function improvement	[77]
		-improved oocyte/embryo quality and pregnancy rates	[81]
		-restoration of ovarian proper histological features, TGR ratio and fertility in mice	[87]
**Metabolic abnormalities**	**MI**	-improved glucose-to-insulin ratio and HOMA index	[63]
		-increased circulating HDL level, weight loss, and leptin reduction	[98]
	**DCI**	-improved insulin resistance, blood pressure, and plasma TG concentration	[65]
		-better insulin sensitivity, BMI reduction	[62]
	**both**	-body weight and insulin level reduction	[69]
		-reduced fasting insulin and HOMA index	[59]
		-improved LDL, HDL, and TG levels, HOMA index reduction	[99]

T—testosterone, E2—estradiol, SHBG—sex hormone-binding globulin, P4—progesterone, FSH—follicle stimulating hormone, LH—luteinizing hormone, TGR—ratio of theca/granulosa cell layer thickness, HOMA—Homeostatic Model Assessment, BMI—body mass index, LDL—low-density lipoprotein cholesterol, HDL—high-density lipoprotein cholesterol, TG—triglycerides.
